# Alcohol-based hand rub and incidence of healthcare associated infections in a rural regional referral and teaching hospital in Uganda (‘WardGel’ study)

**DOI:** 10.1186/s13756-017-0287-8

**Published:** 2017-12-28

**Authors:** Hiroki Saito, Kyoko Inoue, James Ditai, Benon Wanume, Julian Abeso, Jaffer Balyejussa, Andrew Weeks

**Affiliations:** 1 Japan Ministry of Health, Labour and Welfare, Health Bureau, Tokyo, Japan; 2 Institute of Tropical Medicine, Nagasaki University, Nagasaki, Japan; 3 Sanyu Africa Research Institute, Mbale, Uganda; 4 Mbale Regional Referral Hospital, Departments of Community Medicine, Paediatrics and Surgery, Mbale, Uganda; 5 University of Liverpool, Sanyu Research Unit, Liverpool, UK

**Keywords:** Healthcare epidemiology, Infection prevention and control, Alcohol-based hand rub

## Abstract

**Background:**

Good hand hygiene (HH) practice is crucial to reducing healthcare associated infections (HAIs). Use of alcohol-based hand rub (ABHR) at health facilities is strongly recommended but it is limited in Uganda. Data on the practice of HH and the incidence of HAIs is sparse in resource-limited settings. We conducted a quasi-experimental study to evaluate HH practices of health care providers (HCPs) utilizing locally made ABHR and the incidence of HAIs.

**Methods:**

HH compliance among HCPs and the incidence of HAIs were assessed at Mbale Regional Referral Hospital, a teaching hospital in rural Uganda. Inpatients from the obstetrics/gynecology (OBGYN), pediatric and surgical departments were enrolled on their day of admission and followed up during their hospital stay. The baseline (pre-intervention) phase of 12-weeks was followed by a 12-week intervention phase where training for HH practice was provided to all HCPs present on the target wards and ABHR was supplied on the wards. Incidence of HAIs and or Systemic Inflammatory Response Syndrome (SIRS) was measured and compared between the baseline and intervention phases. Multivariate survival analysis was performed to identify associated variables with HAIs/SIRS.

**Results:**

A total of 3335 patients (26.3%) were enrolled into the study from a total of 12,665 admissions on the study wards over a 24-week period. HH compliance rate significantly improved from 9.2% at baseline to 56.4% during the intervention phase (*p* < 0.001). The incidence of HAIs/SIRS was not significantly changed between the baseline and intervention phases (incidence rate ratio (IRR) 1.07, 95% CI: 0.79 – 1.44). However, subgroup analyses showed significant reduction in HAIs/SIRS on the pediatric and surgical departments (IRR 0.21 (95% CI: 0.10 – 0.47) and IRR 0.39 (95% CI: 0.16 – 0.92), respectively) while a significant increase in HAIs/SIRS was found on the OBGYN department (IRR 2.99 (95% CI: 1.92 – 4.66)). Multivariate survival analysis showed a significant reduction in HAIs/SIRS with ABHR use on pediatric and surgical departments (adjusted hazard ratio 0.26 (95% CI: 0.15 – 0.45)).

**Conclusions:**

To our knowledge, this study is one of the largest studies that address HAIs in Africa. During the 24-week study period, significant improvement in HH compliance was observed by providing training and ABHR. The intervention was associated with a significant reduction in HAIs/SIRS on the pediatric and surgical departments. Further research is warranted to integrate HAIs surveillance into routine practice and to identify measures to further prevent HAIs in resource limited settings.

**Trial registration:**

ClinicalTrials.gov NCT02435719, registered on 20 April, 2015 (retrospectively registered).

## Background

Hand hygiene (HH) is a basic yet critical practice to prevent healthcare associated infections (HAIs) [1]. However, studies have been conducted mostly in high-income countries and little is known about HH practice and HAIs in the resource limited settings [2]. Alcohol-based hand rub (ABHR) has been recommended over hand washing with soap and water by the World Health Organization (WHO) because of its wide microbiological spectrum, time efficiency, availability at the point of care, and improved skin tolerance [2]. Local production of ABHR has been shown to be feasible globally, even in low- and middle-income countries (LMICs) [3]. However, multiple other behavioral, cultural and religious factors also need to be considered in HH improvement programs.

The current high disease burden of HAIs and their preventability have led to a global emphasis on HAI prevention in the face of antimicrobial resistance as described in Global Health Security Agenda and WHO global action plan on antimicrobial resistance [4, 5]. In the USA, for example, HAI prevention has been included as one of the national health objectives [6]. However, the studies on HAIs from LMICs are limited, variable, and often of poor quality, and mainly focused on a single disease entity such as surgical site infection (SSI) [7–9]. Moreover, the association between HH improvement, especially with use of ABHR, and HAIs reduction has been rarely described in the resource limited setting. We therefore carried out a clinical study with the following aims in Uganda:

1. To assess the baseline HH practice among health care providers (HCPs) and the impact of ABHR and training in its use on the HH practice improvement
2. To determine the incidence of HAIs and the effectiveness of ABHR on the reduction of HAIs

## Methods

### Study design and setting

We conducted a quasi-experimental study (named as the ‘WardGel’ study) in which HH compliance and HAI rates were compared before and after the introduction of ABHR on 3 clinical departments in Mbale Regional Referral Hospital (MRRH), a government hospital in eastern Uganda. MRRH is one of the 14 governmental regional referral hospitals in Uganda, and serves over four million people in its catchment area of 15 local districts and beyond. The hospital has 12 wards with 550 beds. It also functions as a teaching hospital where there are 40 physicians (including interns), 170 nurses and students including medical students and nursing students. Before this study, ABHR was only used by a small number of senior HCPs such as nursing supervisors who carried portable ABHR bottles. This was mainly because of financial constraints that prevented its purchase by the hospital. As such, ABHR use was almost non-existent prior to this study. Typically, only one or no functional sinks/taps were available in each ward. Portable water bottles and basins were an alternative for hand washing. Gloves, even non-sterile ones, were rarely available. A ward was usually a single, open space without isolation rooms. The hospital followed a standard operating procedure for general environmental cleaning, which was unchanged during the study period.

We selected five wards across three departments as the study sites, namely; the acute and general pediatric wards (pediatric department), the gynecology and post-natal wards (obstetrics/gynecology (OBGYN) department), and the general surgical ward (surgical department). The two pediatric wards were selected because evidence of the impact of ABHR on HAIs among pediatric patients has been particularly scarce in resource limited settings [7]. The latter three were selected because there were relatively few cases of infection on admission on those wards, and it was thought that HAIs would be more accurately observed than at other wards where febrile illnesses such as malaria were more common on admission. Overall, the study was designed so that a wide variety of the patients were observed for multiple types of HAIs to enhance generalisability of the study.

The study was conducted over 24 weeks between October 2014 and April 2015, with the first 12 weeks of the baseline (pre-intervention) phase followed by 12 weeks of the intervention phase. There was a 4-week study interruption between December 2014 and January 2015 (after week 8) due to the holiday seasons in Uganda when the number of inpatients and HCPs were low. Inclusion criteria for the study were all the patients who were admitted on the above selected wards during the study period (hospital day 1 = day of admission).

During the baseline phase, the HH compliance rate among HCPs was assessed by direct observations performed by the trained research assistants on the wards, based on the WHO hand hygiene technical reference manual and the WHO five moments for HH, i.e. before touching a patient, before clean/aseptic procedure, after body fluid exposure risk, after touching a patient, and after touching patient surroundings [2]. Each trained research assistant on each ward observed HCPs during the day-times only for targeted ward activities including busy times such as ward rounds by physicians. The research assistants conducted observations openly, without interfering with the ongoing clinical work, but kept the identity of the HCP confidential, observing up to a maximum of three HCPs simultaneously provided there were no missing opportunities. One observation session lasted for 10-30 min; and only prolonged the sessions in situations of observing a care sequence to its end.

The HH compliance was calculated by the number of observed HH actions (using either ABHR or hand washing with soap and water) upon an opportunity divided by the total number of opportunities for HH actions. The amount of ABHR consumption from non-portable bottles was assessed as a supplemental indication for HH compliance through ABHR. The incidence of HAIs was also measured during the baseline phase through prospective follow-up of patients by research assistants (see below).

During the intervention phase, in addition to the study activities performed during the baseline phase, the one-litre ABHR bottles fitted in a locally-made metallic holder were mounted on the walls of the wards where the access to ABHR was thought to be convenient. Mobile bottles were also placed on the trolleys for ward rounds, on the reception area, and on the treatment area. Portable 40 ml hand-sized bottles were also provided to HCPs and kept available throughout the intervention phase. The ABHR used in the study was Alsoft V, ABHR locally made from sugar cane in Uganda by Saraya East Africa Co. Ltd. It manufactured locally, but according to international standards (Good Manufacturing Practice), and contained the recommended concentration of ethanol (76.9 to 81.4 vol%) [2, 10, 11]. An introductory training session on ABHR use was provided to all the HCPs on the target wards by the research team in week 12 with the help of staff trainers from Saraya East Africa Co. Ltd. In order to maintain the HH compliance, the introductory training was followed by the distribution of educational posters on HH in week 17 and follow-up training in week 18. Additional training was also conducted as required when new medical students and nursing students came to the wards. As the first training session was provided in the last week of the baseline phase, patients who were hospitalized in week 12 and week 13 were excluded from the final analysis of the incidence of HAIs to minimize study contamination between the baseline and the intervention phases.

For the assessments, a paper-based surveillance form was created to record demographics, patient interventions (e.g. surgical interventions), vital signs, clinical findings, antibiotic use and patient’s outcomes. SSIs, urinary tract infections (UTIs), pneumonia, central nervous system (CNS) infections, gastroenteritis and episiotomy infections were selected as the HAIs measured in this study. Each definition of HAIs was modified from the 2014 version of United States Center for Disease Control and Prevention’s National Healthcare Safety Network (CDC/NHSN) surveillance definitions of HAIs, considering locally available resources [12]. Research assistants (mainly registered nurses but with one physician) were trained to fill out the surveillance form, to measure vital signs, to identify relevant clinical signs from patients’ medical records, and to record laboratory and imaging findings. After enrollment into the study, the patients were prospectively followed on a daily basis until discharge. Post-discharge follow-up calls were also attempted for all the patients around 1 month after their discharge. Individual patients’ data collected from patients’ medical records and vital signs measured by the research assistants were also reviewed by research supervisor for quality assurance of the data. All the data collected on the paper-based forms were entered into an Epi Info® database installed onto computers at the Sanyu Africa Research Institute (SAfRI). The only exception was the HH compliance data that was entered into the WHO-produced Microsoft Word® data collection sheet for analysis [13].

### Outcomes

The primary outcomes were the HH compliance rates among HCPs and the incidence rates of HAIs before and after the intervention. The secondary outcomes included antibiotic usage, length of hospital stay and hospital mortality of the study participants.

During the run-in period, it was noted that medical documentation by physicians and clinical officers was not always sufficient to make a diagnosis of an HAI as defined for this study. Direct questioning of the physicians and clinical officers involved with the patient management was also difficult due to their busy schedule and difficulty in recall given the large volume of patients seen per physician. There was therefore a post-hoc change in the primary outcome from HAIs only to the composite outcome of HAIs and or criteria for systemic inflammatory response syndrome (SIRS) occurring on hospital day 3 or after (SIRS/HAI) (c.f. SIRS criteria for adult patients, two or more of: 1. temperature > 38 °C or <36 °C, 2. heart rate > 90/min, 3. respiratory rate > 20/min or Paco_2_ < 32 mmHg, 4. white blood cell (WBC) count >12,000/mm^3^ or <4000/mm^3^ or >10% immature bands. SIRS criteria for pediatric patients, two or more of the same four items with variable thresholds for age, at least one of which must be temperature or WBC) [14, 15]. Those with SIRS on hospital day 1 or 2 of their hospital stay were excluded from the composite primary outcome. Paco_2_ and WBC were rarely performed at MRRH, therefore vital signs measured by research staff were mostly used to determine whether SIRS criteria was met or not.

### Statistical analysis

Means, standard deviations (SD) with t-tests, and proportions with chi-squared tests were calculated for continuous and categorical variables in bivariate analyses, respectively, in order to describe demographics and clinical variables of the study participants. Poisson regression analysis was used to compare HH compliance rates before and after the intervention. Linear regression analysis was used to compare HH compliance rate and ABHR consumption during the intervention. Relative risks and incidence rate ratios of SIRS/HAI were also calculated to compare risks and incidence rates before and after the intervention, respectively. Survival analyses with the cox proportional hazard model were performed to calculate hazard ratios (HRs) in order to describe the associated variables for SIRS/HAI. Multivariate survival analysis with backward selection and plausible causal interpretation was used to calculate adjusted HRs. Statistical significance was defined as a *p* value of <0.05 and 95% confidence intervals (CIs) were reported. All statistical analysis was conducted using Statistical Analysis System (SAS®) version 9.3 (SAS Institute, Cary, NC, USA).

There was no formal HAI rate reported in Uganda that we could use for the sample size calculation. We therefore used the WHO data for developing countries to estimate that 10% of the hospitalized patients would develop any type of HAI [16]. We estimated a 3% reduction in HAIs after the intervention. With the level of significance defined as α = 0.05, and statistical power as β = 0.80, we estimated 1356 patients each would be required before and after the intervention.

### Role of the funding source

The study was conducted with funding provided by Saraya East Africa Co. Ltd., who also provided the ABHR made at their local factory in Uganda. Their staff helped the research team conduct HH training on the wards. Otherwise, the funder was not involved in the study design, data collection, data analysis, data interpretation, or manuscript writing.

The study was approved by the Mbale Regional Hospital Institutional Review Committee (MRHIRC)(REIRC IN – COM 098/2014) and registered to ClinicalTrials.gov (NCT02435719).

## Results

### Hand hygiene compliance

**Fig. 1:**
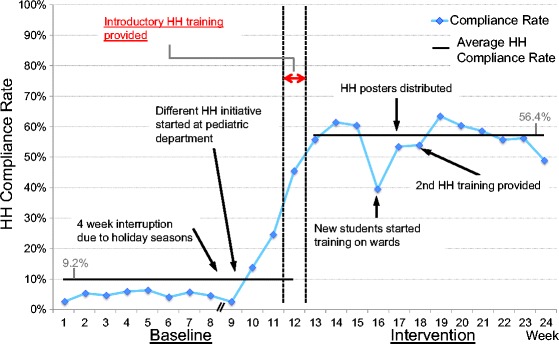
Overall Hand Hygiene (HH) Compliance Before and After Intervention. x-axis: study week (Week 1-12: Baseline, Week 13-24: Intervention). y-axis: HH compliance rate (%). light blue line: HH compliance rate

**Fig. 2:**
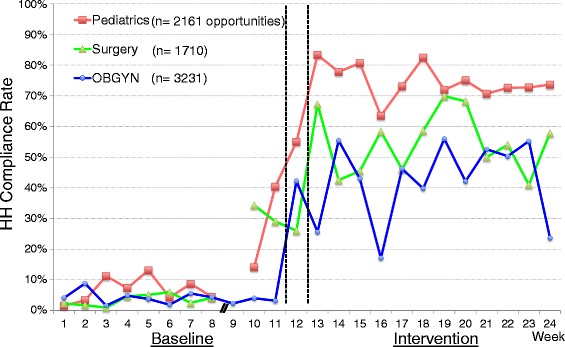
Hand Hygiene (HH) Compliance by Department. x-axis: study week (Week 1-12: Baseline, Week 13-24: Intervention). y-axis: HH compliance rate (%). red line: Pediatrics. green line: Surgery. blue line: OBGYN

**Fig. 3:**
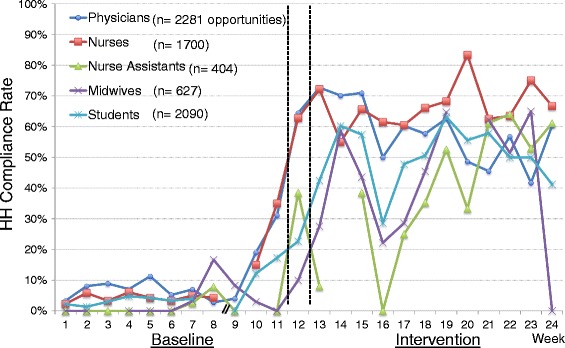
Hand Hygiene (HH) Compliance by Profession. x-axis: study week (Week 1-12: Baseline, Week 13-24: Intervention). y-axis: HH compliance rate (%). blue line: Physicians. red line: Nurses. green line: Nurse Assistants. purple line: Midwives. light blue line: Students

In total, 7102 HH opportunities were observed (3770 and 3332 opportunities in baseline and intervention phases, respectively). HH compliance rate remained very low for most of the baseline weeks (Fig. 1). The overall compliance rate from week 1 to week 9 was 4.6%. During week 10, a neonatal unit was opened in one section of the pediatric wards. As part of that initiative, the pediatricians provided HH education (without ABHR provision). All staff on the unit were requested to wash their hands with soap and water before entering the neonatal unit and touching the patients. In addition, educational posters were placed at the entrance and the patient registration area of the unit. As a result, the HH compliance rate rose on the pediatric wards, starting with an increased rate in week 10. There is evidence that there was higher compliance elsewhere in the hospital with the surgical department also showing higher rates starting in week 10. During week 12, when the introductory HH training was provided along with portable ABHR bottles for HCPs by our study group, the HH compliance rate continued to rise steadily. The average HH compliance rate during the intervention phase (week 13 through 24) was significantly higher compared with that during the baseline (56.4% vs. 9.2%, rate difference 47.2, 95% CI 44.5-50.0, *p* < 0.001 (Poisson regression)). During the intervention phase, the HH compliance rate dropped once in week 16 when a large group of new medical students and nursing students started clinical rotations on the wards. The effect was seen amongst the new students, but also seen in other cadres of staff during that week. However, the rate increased after the posters for HH promotion and second HH training were provided during week 17 and 18, respectively, and remained at the similar level thereafter. The HH compliance rates by department during the intervention phase were 75.9% at the pediatric department, 54.4% at the surgical department, and 44.1% at the OBGYN department, respectively (*p* < 0.001 (chi-square)), and the HH compliance rate during the intervention phase was significantly lower at the OBGYN department than the other departments (44.1% vs. 67.6%, rate difference 23.5%, 95% CI 20.3-26.8, *p* < 0.001 (chi-square)) (Fig. 2). The HH compliance rates by profession during the intervention phase were 66.0% among nurses, 61.0% among physicians, 51.5% among midwives, 50.6% among students, and 46.9% among nurse assistants, respectively (p < 0.001 (chi-square); Fig. 3). During the intervention phase, the amount of ABHR consumption from non-portable bottles attached on the wards was measured. There was no significant linear correlation between HH compliance rate and ABHR consumption (*r* = −0.27, *p* = 0.39 (Pearson correlation)).

### Baseline characteristics of the study participants

There were 12,665 admissions across the selected five wards during the entire study period. From these, a total of 3335 patients (26.3%) were enrolled into the study, excluding those who were hospitalized in study weeks 12 and 13 from the analyses. Enrollment of patients into the study was limited by the availability of research staff who only attended the wards once a day to collect data. Those patients who were admitted and discharged between their visits were therefore missed by the data collectors. The 1723 (51.7%) adult patients had a mean age of 29.7 years whilst the 1612 (48.3%) pediatric patient had a mean age of 3.9 years (Table 1). Patients in the OBGYN department composed 47.8% of the study patients with the post-natal ward being the largest of the five wards studied. During their hospitalization 2286 (68.6%), 573 (17.3%), and 873 patients (26.3%) received antibiotic therapy, urinary catheter placement, and surgery respectively. Two hundred ten patients (6.3%) required mechanical ventilation, but virtually all of these (208; 99.0%) required it peri-operatively on the day of surgery only. Although there were relatively few pediatric patients and an excess of surgical patients enrolled into the study during the intervention phase, the rest of the patient characteristics were comparable between the baseline and intervention phases (Table 1).

**Table 1:** Characteristics of enrolled patients

	All *n* (%)	Baseline *n* (%)	Intervention *n* (%)	*p* value
Number of patients enrolled	3335	1848 (55.4)	1487 (44.6)	
Adult	1723 (51.7)	898 (48.6)	825 (55.5)	<0.01
Female	2369 (71.0)	1290 (69.8)	1079 (72.6)	0.08
Mean age among adult patients, (SD), y	29.7 (12.6)	29.4 (11.9)	30.1 (13.3)	0.21
Mean age among pediatric patients, (SD), y	3.9 (5.0)	3.8 (5.0)	4.1 (4.9)	0.24
Department				
OBGYN	1595 (47.8)	854 (46.2)	741 (49.8)	<0.01
Pediatrics	1336 (40.1)	814 (44.1)	522 (35.1)	
Surgery	404 (12.1)	180 (9.7)	224 (15.1)	
Ward				
Post-natal	1139 (34.1)	621 (33.6)	518 (34.8)	<0.01
Gynecology	456 (13.7)	233 (12.6)	223 (15.0)	
General pediatrics	669 (20.1)	359 (19.4)	310 (20.8)	
Acute pediatric unit	667 (20.0)	455 (24.6)	212 (14.3)	
Surgery	404 (12.1)	180 (9.8)	224 (15.1)	
Living in rural region	2634 (79.0)	1455 (78.8)	1179 (79.3)	0.74
Education (adult patients only)				
None	786 (45.7)	401 (44.8)	385 (46.7)	0.28
Primary	605 (35.1)	313 (34.9)	292 (35.4)	
Secondary	255 (14.8)	135 (15.1)	120 (14.5)	
Tertiary	75 (4.4)	47 (5.2)	28 (3.4)	
Antibiotic use	2286 (68.6)	1290 (69.8)	996 (67.0)	0.08
Mean length of antibiotic use (SD), d	2.4 (2.6)	2.2 (2.1)	2.7 (3.1)	<0.01
Urinary catheter use	573 (17.3)	323 (17.6)	250 (16.9)	0.58
Mean length of urinary catheter use, (SD), d	2.3 (3.8)	1.3 (1.4)	3.5 (5.2)	<0.01
Mechanical ventilation Use	210 (6.3)	78 (4.3)	132 (8.9)	<0.01
Mean length of mechanical ventilation use, (SD), d	0.0 (0.1)	0.0 (0.2)	0.0 (0.0)	0.07
Surgery performed	873 (26.3)	491 (26.8)	382 (25.7)	0.51
Major surgery	611 (70.0)	339 (69.0)	272 (71.2)	0.49
General anesthesia	324 (37.3)	159 (32.7)	165 (43.3)	<0.01
Prophylaxis antibiotic use	800 (91.6)	443 (90.2)	357 (93.5)	0.09

### Healthcare associated infections

There were 95 (5.1%) and 87 (5.9%) patients who were diagnosed as having HAIs and or met the SIRS criteria during the baseline and intervention phases, respectively (relative risk (RR) of 1.14, 95% CL 0.86-1.51, *p* = 0.37; Table 2). Among the total 182 patients with SIRS/HAI during the whole study period, 178 patients (97.8%) met SIRS criteria whilst 20 patients (11.0%) were diagnosed with at least one of HAIs. Only 4 had a diagnosis of HAIs without meeting the SIRS criteria. The most commonly diagnosed HAI was SSI (12 patients, 0.4%), followed by pneumonia (8 patients, 0.2%), gastroenteritis (1 patient) and episiotomy infection (1 patient). UTI and CNS infections were not diagnosed. The median hospital day of SIRS/HAI incidence was day 3 (range 3-17) whilst the median post-operative day (POD) of SIRS/HAI incidence was day 2 (range 0-12, *n* = 100). For the 87 patients who had a urinary catheter placed and later met the SIRS criteria, SIRS was identified a median of 2 days after catheter placement (range 0-11). Fifty-one patients (58.6%) had a urinary catheter in place when SIRS was identified. For 33 patients who received mechanical ventilation and later developed SIRS and or pneumonia, the incidence of SIRS and or pneumonia occurred a median of 2 days after the start of mechanical ventilation (range 1-9).

**Table 2:** Incidence of systemic inflammatory response syndrome (SIRS) and healthcare associated infections (HAIs)

	All n (%) (*N* = 3335)	Baseline n (%) (*N* = 1848)	Intervention n (%) (*N* = 1487)	Relative risk (RR) or incidence rate ratio (IRR) (95% CI, *p* value)
SIRS incidence since hospital day 3 and/or HAIs diagnosed	182 (5.5)	95 (5.1)	87 (5.9)	1.14 (0.86-1.51, 0.37)
OBGYN	99 (6.2)	27 (3.2)	72 (9.7)	3.07 (2.00-4.73, <0.01)
Pediatrics	58 (4.3)	51 (6.3)	7 (1.3)	0.21 (0.10-0.47, <0.01)
Surgery	25 (6.2)	17 (9.4)	8 (3.6)	0.38 (0.17-0.86, 0.01)
SIRS criteria met	178 (5.4)	91 (5.0)	87 (5.8)	1.18 (0.89-1.57, 0.25)
Diagnoses of HAIs made	20 (0.6)	13 (0.7)	7 (0.5)	0.67 (0.27-1.67, 0.39)
Surgical site infection^a^	12 (0.4)	6 (0.3)	6 (0.4)	1.24 (0.40-3.84, 0.71)
Pneumonia^a^	8 (0.2)	8 (0.4)	0 (0.0)	0 (, 0.01)
Gastroenteritis^a^	1 (0.03)	1 (0.05)	0 (0.0)	0 (, 0.55)
Episiotomy infection^a^	1 (0.03)	1 (0.05)	0 (0.0)	0 (, 0.55)
Incidence rate of SIRS/HAI (cases/1000 patient-days)	18.0	17.4	18.6	1.07 (0.79–1.44, 0.66)
OBGYN	25.6	13.4	40.1	2.99 (1.92–4.66, <0.01)
Pediatrics	15.0	21.7	4.6	0.21 (0.10–0.47, <0.01)
Surgery	10.2	15.8	6.2	0.39 (0.16–0.92, 0.02)
SIRS/HAI incidence per 100 surgeries (*n* = 873)	11.5	7.5	16.5	2.19 (1.49-3.21, <0.01)
OBGYN (*n* = 639)	13.5	7.2	22.5	3.14 (2.05-4.82, <0.01)
Surgery (*n* = 234)	6.0	8.8	3.3	0.38 (0.12-1.18, 0.10)
SIRS incidence per 100 urinary catheter use (*n* = 573)	14.8	7.7	24.0	3.10 (2.00-4.80, <0.01)
OBGYN (*n* = 545)	14.9	7.6	24.1	3.18 (2.02-5.00, <0.01)
Surgery (*n* = 28)	14.3	10.5	22.2	2.11 (0.35-12.67, 0.30)
SIRS/pneumonia incidence per 100 mechanical ventilation (*n* = 210)^b^	15.7	11.5	18.2	1.58 (0.77-3.22, 0.20)
OBGYN (*n* = 112)	24.1	10.4	34.4	3.30 (1.35-8.08, <0.01)
Surgery (*n* = 97)	5.2	10.3	2.9	0.28 (0.05-1.61, 0.16)

^a^ Multiple diagnoses are possible for each individual

^b^ Includes one case each who had mechanical ventilation without surgery at pediatric and OGBYN departments

When stratified by departments, the RR and incidence rate ratio (IRR) showed the incidence of SIRS/HAI was significantly lower during the intervention phase in the pediatric and surgical departments whilst the incidence rose significantly in the OBGYN department (Table 2). The incidence of SIRS/HAI per surgery was also lower in the surgical department during the intervention phase (RR 0.38, 95% CI 0.12-1.18, *p* = 0.10) while it was increased in the OBGYN department (RR 3.14, 95% CI 2.05-4.82, *p* < 0.01). On the other hand, the incidence of SIRS in those with a urinary catheter and the incidence of SIRS and or pneumonia in those who had mechanical ventilation were both significantly higher during the intervention in the OBGYN department (RR for SIRS in those with a urinary catheter 3.18, 95% CI 2.02-5.00, *p* < 0.01; and RR for SIRS and or pneumonia in those receiving mechanical ventilation 3.30, 95% CI 1.35-8.08, *p* < 0.01, respectively) while those at the surgical department were not significantly changed (RR for SIRS per urinary catheter use 2.11, 95% CI 0.35-12.67, *p* = 0.30; and RR for SIRS and or pneumonia per mechanical ventilation 0.28, 95% CI 0.05-1.61, *p* = 0.16, respectively).

Bivariate survival analysis to describe the associated variables for SIRS/HAI showed no overall statistical association between the intervention and the SIRS/HAI incidence (HR 1.10, 95% CI 0.83-1.48, *p* = 0.50; Table 3), but multivariate analysis stratified by department showed the intervention was significantly associated with the lower SIRS/HAI incidence at the pediatric and surgical departments (adjusted HR 0.26, 95% CI 0.15-0.45, *p* < 0.01), after adjusting for gender and occurrence of surgery. Conversely, the intervention was significantly associated with increased SIRS/HAI incidence at OBGYN department (adjusted HR 3.10, 95% CI 1.98-4.84, p < 0.01), after adjusting for occurrence of surgery, prior antibiotic use (including antibiotic prophylaxis for those who received surgery) and mechanical ventilation, the latter two of which were considered confounders for occurrence of surgery.

**Table 3:** Patient characteristics associated with systemic inflammatory response syndrome (SIRS) and healthcare associated infections (HAIs)

	Hazard ratio (HR) of SIRS/HAI (95% CI, *p* value)	Adjusted HR (95% CI, *p* value)	
		Pediatrics or Surgery (*n* = 1740)	OBGYN (*n* = 1595)
Alcohol based hand gel provision and hand hygiene promotion (intervention)	1.10 (0.83-1.48, 0.50)	0.26 (0.15-0.45, <0.01)	3.10 (1.98-4.84, <0.01)
Adult	1.64 (1.22-2.20, <0.01)		^a^
Female	2.11 (1.49-3.00, <0.01)	1.43 (0.93-2.20, 0.11)	^a^
Age among adult patients, y	0.99 (0.98-1.01, 0.24)		
Age among pediatric patients, y	1.02 (0.97-1.06, 0.47)		
Pediatrics/Surgery (vs. OBGYN)	0.40 (0.30-0.54, <0.01)	^a^	^a^
Living in rural region	1.01 (0.70–1.46, 0.94)		
Education – none (adult patients only)	0.93 (0.63-1.35, 0.69)		
Surgery performed prior to SIRS/HAI	1.80 (1.34-2.41, <0.01)	0.57 (0.31-1.04, 0.07)	1.42 (0.75-3.00, 0.28)
Antibiotic used prior to SIRS/HAI	1.85 (1.05–3.25, 0.03)		4.99 (1.47-16.98, 0.01)
Urinary catheter used prior to SIRS/HAI	2.63 (1.96-3.53, <0.01)		
Mechanical ventilation used prior to SIRS/HAI	1.59 (1.09-2.34, 0.02)		1.53 (0.97-2.42, 0.07)

^a^Variables were not used because they were completely discrete in the models

The incidence of SIRS/HAI was significantly associated with higher hospital mortality (RR11.55, 95% CI 4.78-27.93, p < 0.01), longer length of hospital stay (mean difference 3.8 days, 95% CI 3.4-4.2, p < 0.01), and longer duration of antibiotic use (mean difference 2.3 days, 95% CI 1.9-2.7, p < 0.01)(Table 4).

**Table 4:** Clinical outcomes associated with systemic inflammatory response syndrome (SIRS) and healthcare associated infections (HAIs)

	All n (%) (*N* = 3335)	SIRS/HAI n (%) (*N* = 182)	No SIRS/HAI n (%) (*N* = 3153)	Relative risk (RR) (95% CI, *p* value) or *p* value of t-test
Hospital mortality	20 (0.6)	8 (4.4)	12 (0.4)	11.55 (4.78-27.93, <0.01)
Left against medical advice	395 (11.8)	17 (9.3)	378 (12.0)	0.78 (0.49-1.24, 0.28)
Mean length of stay, (SD), d	2.7 (3.0)	6.3 (6.7)	2.5 (2.5)	<0.01
Mean length of antibiotic use, (SD), d (*n* = 2286)	2.4 (2.6)	4.5 (5.6)	2.2 (2.1)	<0.01

During the entire study period, there were only 14 blood cultures sent (0.4% of the total 3335 enrolled patients), 12 swab wound cultures sent (0.3%), and one X-ray performed. Other relevant laboratory and imaging tests such as urine cultures and gram stain of cerebrospinal fluid were never performed.

## Discussion

HAIs are a serious but often overlooked problem in many LMICs. Introduction of locally made ABHR with HH education significantly improved HH practice at this regional teaching hospital in rural Uganda, leading to a significant reduction in the incidence of HAIs and or SIRS on the pediatric and surgical services. In contrast, however, there was an increase in SIRS/HAI on the OBGYN wards. The incidence of SIRS/HAI was found to be associated with adverse clinical outcomes such as higher hospital mortality, longer length of hospital stay and longer length of antibiotic use. We believe therefore that ABHR use with improved HH practice can have a significant impact both at the individual level for patients’ health and at the hospital level in resource limited settings where hospitals are commonly over-crowded and drug supplies are limited and unstable.

Our study showed the baseline HH compliance was only 9.2%, lower than previous studies that reported 16.5%, 12%, and 34.1% in the hospital setting in Ethiopia, Ghana and Rwanda, respectively, though there were some differences in the methods used to measure HH compliance rate [17–19]. Before the start of the neonatal unit on the pediatric wards, the HH compliance was as low as 4.6%, implying the HH practice was almost non-existent at worst. However, the ABHR provision and HH education significantly improved the HH compliance to 56.4%, peaking at 75.9% in the pediatric department. A cross-sectional study conducted in Ethiopia showed that the knowledge of HH and availability of ABHR on the ward were associated with better HH practice [17]. Our study confirmed their findings in a quasi-experimental model and showed that impressive improvements in HH are possible through ABHR provision and HH education: we found a 6-fold increase in the HH compliance rate from under 10%. The different HH initiative that started at the pediatric department during our baseline phase accidentally provided an interesting insight: that HH education alone, without ABHR provision, also improved the HH compliance in all departments. In our study, ABHR use was not differentiated from hand washing with soap and water when the compliance rate was measured. Interestingly, the chief surgeon and nurse in the surgical department started using the water basin during inpatient rounds after the hospital infection-prevention committee suggested it, as follow-up from the pediatric initiative. It was also observed that once HCPs began to use ABHR properly on the wards, they remarked that the smell on the wards improved and the number of flies flying around admitted patients on the bed reduced. This led to HCPs encouraging one another to HH practice, and provided the senior nurses with further motivation to promote HH in the ward areas. Thus, the presence of the right champions along with effective HH education can influence the culture and behavior of HCPs beyond the intervention area. Furthermore our study showed the HH compliance rate was improved more among nurses and physicians, and this finding was favorable given that those clinical staff, who are recognized as a senior, can have a powerful influence on the rest of the HCPs [20]. The final extent of the education-only improvements is not known as the ABHR provision started shortly afterwards and resulted in further improved HH across the wards.

The ABHR was widely distributed around the wards according to the HCPs’ wishes. This included placing it in dispensers on the walls and on the trolleys for ward rounds. Even though the amount of ABHR consumption from non-portable bottles was not associated with compliance rate during the intervention, it is possible that the portable bottles provided to each member of the clinical staff were more convenient and more often used than the static bottles as the biggest increase in HH compliance was observed in week 12 when the portable bottles were provided. The HH compliance rate was the highest at the pediatric department where our informal follow-up interviews suggested better ABHR “buy-in” from the staff. On the other hand, the HH compliance rate dropped regardless of profession on week 16 when new students started clinical rotations. It was observed that many HCPs were much busier teaching and supervising the new students, giving lower priority to the HH practice. Therefore, in this setting, it appears to be easy to compromise HH practice early in the implementation when it has yet to become established practice. During the intervention, it was noted that the improved HH practice was mostly due to ABHR use. This suggests that knowledge and attitude alone may be insufficient, and that it is critical to have good access to a product for HH such as ABHR bottles. This is particularly important in resource limited settings where water, soap or clean towels to dry hands may not be available on wards.

With the improvements in HH practice through ABHR provision and HH education, a lower incidence of HAIs and or SIRS was observed in the pediatric and surgical departments. Even though the incidence of this composite outcome was mainly explained by SIRS rather than HAIs, the incidence was significantly associated with critical clinical outcomes such as higher hospital mortality, implying that the obtainment of accurate vital signs by trained staff and identifying SIRS correctly could serve as an intermediate variable when a formal surveillance system of HAIs is not established or reliable.

Surprisingly, our study found that the incidence of HAIs and or SIRS was increased in the OBGYN department following the intervention. There are several possible explanations for this finding. First, construction/partitioning work for the neonatal unit establishment started on part of the post-natal ward on week 13, leading to a higher patient throughput in the rest of the ward. This led to many mothers having to sleep on the floor or in the corridors in the overcrowded environment, which could have potentially exposed many of these mothers to infections. Second, the new interns started clinical rotations in the OBGYN department on week 13, and started learning basic OBGYN skills such as cesarean section. Our intervention didn’t cover labor room where vaginal deliveries are performed, or the OBGYN operating room. Therefore, other important preventative measures, such as good aseptic technique during deliveries or surgery might have been suboptimal and may be areas for improvement in the future. Third, HH compliance in the OBGYN department was significantly lower than in the other two departments, and this could account for the lack of positive effect. Sick leave in several of the head midwives during this period resulted in a lack of overall leadership and support, and this is likely to have contributed to the low compliance rate. These factors clearly demonstrate how the ward provision of ABHR and HH education is not enough on its own to prevent all infections. It needs to be provided alongside clinical leadership, good personal hygiene in the operating and labor rooms, as well as good overall hospital space, facilities and cleanliness to reduce the overall infection risk.

Even though this large study provides further insights into ABHR use and HAIs in the resource limited setting, there are several study limitations. First, this study was conducted at a single location with a simple before-and-after design, mainly due to the financial constraints. However, MRRH has a large catchment area as a referral hospital, and the studied population is considered to be representative of the population of eastern Uganda. Longer monitoring of HH compliance, even after the intervention was stopped, could have provided further insight. Second, the HH compliance rate was measured through direct observation by the research staff. This is likely to have caused spontaneous improvements in practice, also known as the Hawthorne effect [21]. This is a bias resulting from a change in behavior of observed study participants leading to improved outcomes. However, direct observation is still considered the gold standard for monitoring HH practice [2]. The lack of a significant association between ABHR consumption from non-portable bottles and observed HH compliance suggests that measuring product consumption may not be an effective way to measure HH compliance. Third, our composite outcome mostly relied on the incidence of SIRS. There was no standardized surveillance system for HAIs at MRRH, and physicians’ and clinical officers’ documentation was often of insufficient quality to make an accurate diagnosis of HAIs. Our research assistants were capable of measuring accurate vital signs to identify SIRS incidence after training, but for them to assess additional clinical findings would have required further training and would have been impractical given the large patient volume. Patients with HAIs may not always develop abnormal vital signs to meet SIRS, and the SIRS incidence on hospital day 3 may not necessarily result from HAIs. However, our study revealed a clear association between SIRS/HAI and more clinically important clinical outcomes such as mortality and length of hospital stay. We therefore consider the composite outcome to be a practical and realistic measure of HAI in resource-limited settings. An impact of a recent change in the definition of sepsis by Third International Consensus Definitions Task Force (Sepsis-3) including utility of quick Sequential [Sepsis-related] Organ Failure Assessment (qSOFA) score for non-intensive care unit patients on diagnosis and treatment of HAIs in the resource limited setting would merit further research as the study, which was conducted in USA, showed qSOFA was a superior predictor of mortality to SIRS [22–24]. Fourth, the study was further compromised by the low use of laboratory and imaging studies for the accurate diagnosis of HAIs. A study in the USA showed that empiric antibiotic therapy was common even when there were no clinical signs of infections, and that obtaining cultures and imaging were associated with narrowing or discontinuation of antibiotics [25]. This requires increased funding into laboratory services, as well as a change in culture of clinical staff. However, it is critical if the world-wide problem of antimicrobial resistance is to be overcome [5, 25]. Fifth, it was very difficult to conduct follow-up of patients post-discharge given the large catchment area and the limited financial resources for the patients. As a re-visit to the hospital for follow-up was logistically difficult for most, we attempted telephone follow-up. However, some phone contacts were not available and in some, the calls went through to family members who were away from the individual patient. The limited successful follow-up calls suggested that some post-operative patients might have developed SSI after discharge, but the other HAIs were difficult to assess by phone alone. This, and the multiple other sources of community infection, led us to focus on the more immediate outcomes that occurred around the time of hospitalization. This was thought to be a more reliable way to assess the impact of our study intervention. Sixth, we did not provide feedback to HCPs, or involve patients and or family members in HH promotion. These interventions could potentially have further improved HH practice among HCPs [26–28]. In addition, HH among patients’ family members may play a role in HAI incidence, particularly in the setting where they are more involved in patients’ care at health facilities. Lastly, we did not incorporate formal qualitative research into our study. This would have helped to explain some of the facilitators and barriers to ABHR use that affected the success of the intervention. We relied instead on field notes collected by the research staff.

## Conclusion

Our study showed that improved HH practice is feasible in resource limited settings and that provision of locally made ABHR and HH education can result in reduced rates of HAIs, especially if there is effective clinical leadership. However, it needs to be seen within a broad context and is unlikely to be effective unless preventive measures are taken within operating and labor rooms, and the problem of hospital hygiene and overcrowding is addressed.

## Abbreviations

ABHR: Alcohol-based hand rub
CDC/NHSN: United States Center for Disease Control and Prevention’s National Healthcare Safety Network
CI: Confidence interval
CNS: Central nervous system
HAI: Healthcare associated infection
HCP: Health care provider
HH: Hand hygiene
HR: Hazard ratio
IRR: Incidence rate ratio
LMICs: Low- and middle- income countries
MRRH: Mbale Regional Referral Hospital
OBGYN: Obstetrics/gynecology
POD: Post-operative day
qSOFA: quick sequential [Sepsis-related] organ failure assessment
RR: Relative risk
SAfRI: Sanyu Africa Research Institute
SIRS: Systemic inflammatory response syndrome
SSI: Surgical site infection
UTI: Urinary tract infection
WBC: White blood cell
WHO: World Health Organization
